# Prospective randomized study to assess the effect of intramuscular glycopyrrolate on hypotension prevention among elderly patients following subarachnoid block during lower limb surgeries

**DOI:** 10.6026/973206300223067

**Published:** 2026-05-31

**Authors:** Neetesh Gautam, Narayan Hari Sharma, Irfan Khan, Shubham Mittal

**Affiliations:** 1 Department of Anaesthesiology, Gajra Raja Medical College, Gwalior, Madhya Pradesh, India; 2 Department of Anaesthesiology, Janak Hospital, Gwalior, Madhya Pradesh, India

**Keywords:** Glycopyrrolate, spinal anesthesia, hypotension, elderly, subarachnoid block, lower limb surgery

## Abstract

Spinal anesthesia is a preferred technique for lower limb surgeries in elderly patients but often leads to hypotension due to sympathetic blockade and diminished cardiovascular adaptability. Glycopyrrolate, an anticholinergic agent, may mitigate this response by sustaining heart rate and venous return. Hence, this prospective randomized study evaluated 50 elderly patients (≥ 60 years) undergoing elective lower limb surgery under spinal anesthesia, divided into a glycopyrrolate group (0.2 mg IM given 30 minutes pre-block) and a control group without premedication. Hemodynamic parameters were recorded intraoperatively and compared between groups. The glycopyrrolate group showed significantly fewer cases of hypotension, better maintenance of mean arterial pressure and heart rate and a reduced need for vasopressors, without notable adverse effects such as tachycardia or dry mouth. Thus, we show that the pre-anesthetic intramuscular glycopyrrolate is a simple, safe and effective measure to enhance hemodynamic stability and prevent hypotension in elderly patients receiving spinal anesthesia.

## Background:

One of the most widely used regional anesthetic methods in lower limb surgeries is spinal anesthesia (subarachnoid block) because it is fast acting, predictable sensory and motor block, minimal airway manipulation and less post-operative complications as compared to general anesthesia [1]. It is particularly beneficial in geriatric patients since it does not involve the instrumentation of the airways and minimizes pulmonary complications. Nevertheless, spinal anesthesia can often be accompanied by severe hemodynamic changes, especially hypotension and bradycardia, which are still the most serious issues in the practice of geriatric anesthesia [2]. Hypotension due to spinal anesthesia is mainly caused by sympathetic blockade causing vasodilation, reduced systemic vascular resistance and pooling of blood in the venous capacitance vessels [3]. It leads to decreased venous return, less cardiac output and the eventual hypotension. The degree of this effect varies with the height of the block, age of a patient, intravascular volume status and cardiovascular reserve [4]. The elderly patients are especially susceptible due to the fact that aging is linked to decreased baroreceptor sensitivity, diminished autonomic response, a decrease in vascular compliance and diminished cardiac reserve that diminishes their capacity to counteract sudden vasodilation. As a result, hypotension in the post-operative period after spinal anesthesia is indicated to be observed in as many as 6575 percent of older patients who undergo lower extremity surgeries, which is a clinically important perioperative issue [5, 6]. Hypotension caused by spinal anesthesia in elderly patients is not just a temporary hemodynamic condition but may cause severe consequences including myocardial ischemia, cerebral hypoperfusion, renal dysfunction, post-operative delirium and a greater perioperative morbidity [7]. Avoidance of Hypotension is thus a significant anesthetic objective in elderly patients. Several preventive measures have been suggested such as fluid preloading, vasopressor prophylaxis, low dose of local anesthetic and drugs which are designed to affect the autonomic tone. In spite of these precautions, hypotension remains a common phenomenon and therefore the need to adopt other preventive strategies [8].

Glycopyrrolate is a quaternary ammonium anticholinergic agent that is commonly used as a vagolytic, antisialagogue in the practice of anesthesia. In comparison to atropine, glycopyrrolate does not penetrate the blood to the brain in the central nervous system and therefore it does not cause side effects in the central nervous system, which is especially beneficial in the elderly [9]. Its antimuscarinic effect decreases vagal tone and increases heart rate that can help sustain cardiac output and blood pressure after spinal anesthesia. Glycopyrrolate has been suggested as a simple pharmacologic intervention to decreases the occurrence of spinal anesthesia-induced hypotension by inhibiting reflex bradycardia and maintaining hemodynamic stability [10]. A number of studies have investigated the use of glycopyrrolate in the hemodynamic stability of spinal anesthesia. In elderly patients, a randomized controlled trial showed that prophylaxis of intramuscular glycopyrrolate was able to substantially decrease the rate of hypotension, vasopressor need and nausea after spinal anesthesia as compared to placebo [11]. In the same manner, another potential randomized trial comparing elderly patients who received spinal anesthesia showed that intramuscular glycopyrrolate given prior to the block decreased the incidence of hypotension and enhanced intraoperative hemodynamic stability. Such results imply that glycopyrrolate can be a valuable addition to traditional prevention methods [12]. Although showing encouraging results, glycopyrrolate prophylaxis prior to spinal anesthesia is not widely used and evidence on the use of glycopyrrolate prophylaxis is scarce in particular groups of patients like elderly patients who undergo lower limb surgeries. Furthermore, the differences in dosing, administration time and patient differences among studies warrant additional prospective randomized studies to elucidate its efficacy and safety profile [13]. Therefore, it is of interest to evaluate the hemodynamic parameters and the vasopressor requirements before deciding whether glycopyrrolate can be suggested as simple, safe and effective prophylactic intervention to achieve the desired perioperative stability in geriatric population.

## Materials and Methodology:

## Study design:

This study was designed as a prospective randomized controlled trial conducted to evaluate the effect of intramuscular glycopyrrolate in preventing hypotension following subarachnoid block in elderly patients undergoing lower limb surgeries.

## Study setting:

The study was conducted in the Department of Anaesthesiology of a tertiary care teaching hospital after obtaining approval from the Institutional Ethics Committee and written informed consent from all participants.

## Study duration:

The study was carried out over a period of 24 months from June 2023 to May 2025.

## Sample size:

A total of 50 patients were included in the study and randomly allocated into two groups of 25 each.

## Inclusion criteria:

[1] Patients aged 60 years and above

[2] Either gender

[3] ASA physical status I-III

[4] Scheduled for elective lower limb surgery under spinal anesthesia

[5] Patients providing written informed consent

## Exclusion criteria:

[1] Refusal to participate

[2] Contraindication to spinal anesthesia

[3] Known hypersensitivity to glycopyrrolate

[4] Severe cardiac disease (arrhythmias, heart block, unstable angina)

[5] Severe hypertension or hypotension pre-operatively

[6] Patients on beta-blockers or autonomic-acting drugs affecting heart rate response

[7] Infection at the spinal injection site

[8] Coagulopathy or anticoagulant therapy

## Randomization and group allocation:

Patients were randomly assigned using a computer-generated randomization table into:

[1] Group G (Glycopyrrolate Group): Received intramuscular glycopyrrolate 0.2 mg 30 minutes prior to spinal anesthesia.

[2] Group C (Control Group): Received no anticholinergic premedication (or IM normal saline placebo depending on your protocol).

## Pre-anaesthetic preparation:

All patients underwent routine pre-anesthetic evaluation one day prior to surgery. Standard fasting guidelines were followed. Baseline investigations including hemogram, renal function tests, ECG and chest X-ray (if indicated) were reviewed.

## On arrival in the operating room:

[1] IV access secured with 18G cannula

[2] Standard monitors attached (ECG, pulse oximeter, non-invasive blood pressure)

[3] Baseline heart rate, systolic BP, diastolic BP, mean arterial pressure and SpO_2_ recorded

[4] All patients received preloading with Ringer's lactate 10 ml/kg over 15-20 minutes before spinal anesthesia.

## Anaesthetic technique:

With the patient in sitting position and under strict aseptic precautions:

[1] Subarachnoid block was performed at L3-L4 or L4-L5 interspace

[2] Using a 25G Quincke spinal needle

[3] After confirming free flow of CSF, 3 ml of 0.5% hyperbaric bupivacaine was injected slowly

[4] Patients were immediately placed supine with slight head elevation.

## Intraoperative monitoring:

Hemodynamic parameters were recorded at:

[1] Baseline (before spinal block)

[2] Immediately after spinal injection

[3] Every 2 minutes for first 10 minutes

[4] Every 5 minutes thereafter until completion of surgery 

## Parameters recorded:

[1] Heart rate (HR)

[2] Systolic blood pressure (SBP)

[3] Diastolic blood pressure (DBP)

[4] Mean arterial pressure (MAP)

[5] Oxygen saturation

## Definitions:

[1] Hypotension: Fall in systolic BP ≥20% from baseline or SBP <90 mmHg

[2] Bradycardia: Heart rate <50 beats/min

## Management of complications:

[1] Hypotension treated with IV ephedrine 6 mg boluses and fluids

[2] Bradycardia treated with IV atropine 0.6 mg

[3] Nausea/vomiting treated with ondansetron

[4] Oxygen administered if SpO_2_ <94%

[5] Total vasopressor requirement and incidence of adverse effects were recorded.

## Outcome measures:

## Primary outcome:

[1] Incidence of hypotension after spinal anesthesia

## Secondary outcomes:

[1] Heart rate trends

[2] Mean arterial pressure trends

[3] Vasopressor requirement

[4] Incidence of bradycardia

[5] Adverse effects (dry mouth, tachycardia, nausea, vomiting) 

## Statistical analysis:

Data were entered into Microsoft Excel and analyzed using statistical software (SPSS version 25). Continuous variables expressed as mean ± standard deviation. Categorical variables expressed as percentage. Student's t-test used for comparison of continuous variables. Chi-square test used for categorical data. p < 0.05 considered statistically significant

## Results:

A total of 50 elderly patients undergoing lower limb surgeries under spinal anesthesia were enrolled and randomly allocated into: Group G (Glycopyrrolate group): 25 patients & Group C (Control group): 25 patients. Both groups were comparable in baseline characteristics. The mean age, gender distribution and ASA status were similar between groups (p > 0.05), indicating no statistically significant difference. This confirms that the two groups were comparable at baseline and suitable for outcome comparison (Table 1). Hypotension was observed in 42% of patients overall, with a significantly lower incidence in the glycopyrrolate group compared to control (6 vs 15 cases, p = 0.018). Bradycardia occurred in 18% of patients overall, with fewer cases in the glycopyrrolate group (2 vs 7), although this difference was not statistically significant (p = 0.072) (Table 2). Vasopressor requirement was significantly lower in the glycopyrrolate group (20% vs 56%, p = 0.011), demonstrating improved intraoperative hemodynamic stability. Although mild anticholinergic effects such as dry mouth and tachycardia were slightly higher in the glycopyrrolate group, these differences were not statistically significant. Nausea and vomiting were more frequent in the control group, likely secondary to hypotension, but the difference was not statistically significant (Table 3).

## Discussion:

Hypotension induced by spinal anesthesia is one of the most common and clinically important adverse effects in elderly patients having lower-limb surgeries. This population is especially susceptible to sudden sympathetic blockage following subarachnoid block due to age-related autonomic dysfunction, decreased baroreceptor sensitivity and decreased cardiac reserve. Pre-emptive pharmacological measures are thus still necessary to ensure stability of hemodynamics [2, 13]. The intramuscular glycopyrrolate applied prior to the spinal anesthesia in the current study had a significant effect on the reduction of the occurrence of hypotension, vasopressor use as well as intraoperative adverse events relative to placebo. These results are in line with previous randomized trials testing the use of glycopyrrolate as a prophylactic agent [6, 9]. A randomized controlled trial by Piya *et al.* [14] showed that prophylactic glycopyrrolate was a significant hypotension prevention in elderly patients undergoing spinal anesthesia (27.3 versus 70.0, p = 0.001) and also decreased the vasopressor demand and nausea. This is in line with the hemodynamic protective effect in our study. Equally, a would-be randomized trial of elderly TURP patients demonstrated that intramuscular glycopyrrolate lowered the occurrence of hypotension (38.7 versus 74.2) and lowered the intravenous fluid and vasopressor needs. These findings support the importance of glycopyrrolate in maintaining circulatory responses following spinal block [15]. Obstetric population studies have demonstrated positive effects as well. A randomized trial of cesarean section patients noted that glycopyrrolate decreased the intensity of hypotension and vasopressor consumption during spinal anesthesia. In spite of the fact that the population is different, the process, which is the attenuation of the vagal predominance and the enhanced cardiac output, is applicable to older surgical patients [7]. Even more recent randomized clinical trials with prophylactic glycopyrrolate prior to spinal anesthesia showed less incidence of hypotension with better hemodynamic stability, but some studies showed no significant difference in vasopressor need. This is an indication that the extent of benefit can be dose-dependent, patient population-dependent and protocol-dependent (anesthetic) [5]. The physiological explanation of the protective effect of glycopyrrolate is its anticholinergic effect, preventing vagally mediated bradycardia and aiding in sustaining cardiac output during sympathetic blockage. It does not cross the blood brain barrier and thus it has fewer central side effects as compared to atropine but offers stable cardiovascular effects [15]. The decrease in hypotension and bradycardia in the current study had a clinical impact in the number of nausea, vomiting and vasopressor events that are clinically significant in older patients in whom hypotension may result in myocardial ischemia, stroke, or renal hypoperfusion. In general, our results are in line with the available literature and show that prophylactic glycopyrrolate is a cheap, easy and efficient approach to hypotension caused by spinal anesthesia to elderly patients who are undergoing lower limb surgeries.

## Conclusion:

We show that intramuscular glycopyrrolate is effective in both prevention and incidence of hypotension in elderly patients who are undergoing lower-limb surgeries. The drug also reduces vasopressor demand and gains intraoperative hemodynamic stability with minimal adverse effects.

## Figures and Tables

**Table 1:** Demographic and baseline characteristics

**Parameter**	**Group G (n=25)**	**Group C (n=25)**	**Percentage (%)**	**p-value**
Mean age (years)	67.2 ± 5.4	66.8 ± 6.1	-	0.78
Male	16	15	62%	0.77
Female	9	10	38%	0.77
ASA I	11	12	46%	0.89
ASA II	10	9	38%	0.89
ASA III	4	4	16%	1

**Table 2:** Incidence of hypotension and bradycardia

**Parameter**	**Group G (n=25)**	**Group C (n=25)**	**Percentage (%)**	**p-value**
Hypotension present	6	15	42% overall	0.018
Hypotension absent	19	10	58% overall	-
Bradycardia present	2	7	18% overall	0.072
Bradycardia absent	23	18	82% overall	-

**Table 3:** Vasopressor requirement and adverse effects

**Parameter**	**Group G (n=25)**	**Group C (n=25)**	**Percentage (%)**	**p-value**
Vasopressor required	5	14	38% overall	0.011
No vasopressor needed	20	11	62% overall	-
Dry mouth	4	1	10% overall	0.15
Tachycardia	3	1	8% overall	0.29
Nausea/Vomiting	2	6	16% overall	0.12
